# Natural language processing-driven knowledge graphs for transformative public health intelligence and research datasets in urgent and emergency care

**DOI:** 10.3389/fpubh.2026.1901339

**Published:** 2026-07-29

**Authors:** Chris Humphries, Atul Anand, Arlene Casey, Wendy Chapman, Kathy Harrison, James M. Hillis, Edward James, Donald J. MacIntyre, Kenneth Nicholson, Lisa Schölin, R. Andrew Taylor, Christopher Tomlinson, David Robertson

**Affiliations:** 1 Generative AI Laboratory, School of Informatics, University of Edinburgh, Edinburgh, United Kingdom; 2 Centre for Cardiovascular Science, University of Edinburgh, Edinburgh, United Kingdom; 3 Centre for Population Health Sciences, University of Edinburgh, Edinburgh, United Kingdom; 4 Clinical Informatics Center, University of Texas Southwestern Medical Center, Dallas, TX, United States; 5 Harvard Medical School, Boston, MA, United States; 6 Mass General Brigham AI and Department of Neurology, Mass General Brigham, Boston, MA, United States; 7 Department of Emergency Medicine, NHS Lothian, Edinburgh, United Kingdom; 8 Centre for Sustainable Delivery, Golden Jubilee National Hospital, Glasgow, United Kingdom; 9 Public Health Scotland, Edinburgh, United Kingdom; 10 Department of Emergency Medicine, University of Virginia, Charlottesville, VA, United States; 11 Department of Biostatistics & Health Informatics, King’s College London, London, United Kingdom; 12 Institute of Health Informatics, University College London, London, United Kingdom; 13 School of Informatics, University of Edinburgh, Edinburgh, United Kingdom

**Keywords:** data interoperability, electronic health records, emergency care, knowledge graph, natural language processing

## Abstract

Urgent and emergency care generates large volumes of clinical data, yet much of the information most useful for research, surveillance, and service planning is recorded only in free-text clinical notes and never reaches the structured datasets used for analysis. As a result, the reasons patients present, the upstream pressures driving demand, and the care that was needed but unavailable remain largely invisible, with consequences that fall most heavily on people who move repeatedly across services. Existing initiatives connect previously separate records or predict future events, but neither recovers the clinical reasoning that explains why people reach crisis. In this Perspective we propose a three-layer knowledge graph architecture for urgent and emergency care: a first layer linking individual contacts into connected patient trajectories across services and time; a second recovering clinical detail lost to reductive coding; and a third capturing the reasoning behind each presentation, including barriers to care and intended but unavailable services. We describe why recent advances in natural language processing and artificial intelligence make this architecture achievable, assess the current evidence - strong for each component, though the integrated architecture awaits validation - and set out the validation, governance, and infrastructure that implementation would require. The architecture offers a route from data volume to usable knowledge, and a means of making patterns of unmet need legible to those who plan and govern care. The constituent technologies now exist; whether this information remains invisible is increasingly a question of design rather than capability.

## Introduction

Urgent and emergency care (UEC) - encompassing emergency departments (EDs), ambulance services, telephone triage, urgent treatment centres, and out-of-hours primary care - generates large volumes of clinical data, yet the information most needed for research and service planning remains inaccessible. Why patients present, what upstream pressures drove each contact, and what care was intended but unavailable are documented in free-text clinical narratives that seldom enter any queryable dataset. This gap between data volume and data utility constrains all UEC research. Its consequences are most visible in populations whose care spans multiple services: patients navigating co-occurring mental health, substance use, and social crises are among the most frequent users of emergency services, but are among the least visible in structured data (1–5). The same is true of frail older patients, whose sentinel events cascade across community, ambulance, emergency, and social care boundaries, with direct health equity consequences (6).

Three compounding structural challenges contribute to the current state. First, organisational fragmentation: patient journeys span multiple organisations, each holding its fragment in a separate database, with cross-organisational linkage dependent on shared identifiers and governance agreements that remain unevenly established (7–9). Second, representational impoverishment: classification systems mandate single primary diagnoses, flattening presentations that span overlapping biopsychosocial domains into reductive codes that lose co-occurring conditions, social context, and clinical complexity (10, 11). Third, inferential invisibility: the clinical reasoning behind each presentation - why the patient presented, what alternatives were attempted, what care was intended but unavailable - is largely captured in free-text narratives that no system interrogates at scale (11). Relational database architectures compound these problems, constraining queries to schemas established at creation and requiring bespoke pipelines for each new research question (12, 13).

Calls from within the UEC community to address these data deficits are growing (11). Consolidated electronic health record systems increasingly span multiple care settings, and interoperability standards such as Fast Healthcare Interoperability Resources (FHIR) are connecting previously siloed data (14, 15). These are necessary but insufficient: linking an ambulance record to an ED attendance cannot answer why the patient called an ambulance or what alternatives they attempted. Those answers require a different form of knowledge representation - one in which relationships between episodes, the reasoning within them, and the intended but unavailable care pathways are explicitly modelled as queryable structure.

In this article, we propose a specific architectural solution: a three-layer knowledge graph designed to link contacts into patient trajectories, recover structured clinical information from free text, and capture the clinical reasoning that existing systems discard. We outline why this architecture is now feasible, what it enables, and how it could be implemented.

## A three-layer knowledge graph architecture

A knowledge graph stores facts as entities connected by named, typed relationships, making the connections between data explicit and directly queryable (16). This structure reflects how clinicians reason about complex patients: an emergency physician seeing a frequent attender thinks not in diagnostic codes but in connections - unstable housing worsening depression, reducing medication adherence, driving the recurrent acute presentations. Current data systems cannot represent this chain; a knowledge graph can, at population scale. Where a relational database records that a patient attended the ED on a given date with a diagnostic code, a knowledge graph represents that an older patient’s attendance after a fall followed a primary care contact at which no acute cause had been found for dizziness. The graph encodes that the primary care contact was preceded by a carer’s report of recurrent falls and reduced eating, and occurred in the context of functional decline documented across earlier contacts (16, 17). Consequently, by learning patterns at population level, the sequence of care contacts predicting health crisis becomes identifiable.

**Figure 1:**
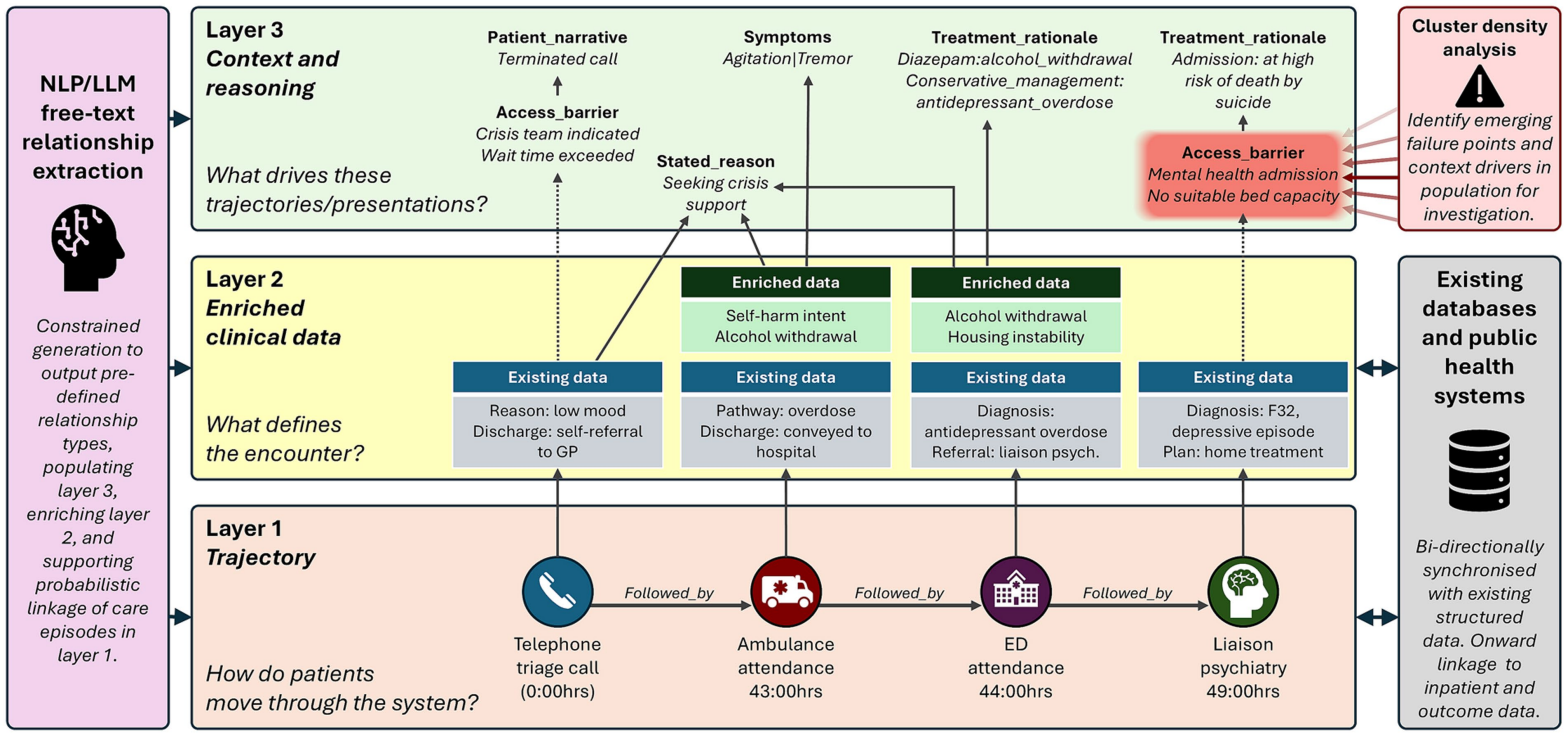
Three-layer knowledge graph architecture for urgent and emergency care, illustrated with a single patient trajectory. Layer 1 (trajectory) links care contacts in sequence. Layer 2 (enriched clinical data) supplements existing structured data with NLP/LLM-extracted clinical entities - recovering self-harm intent, alcohol withdrawal, and housing instability that existing codes do not capture. Layer 3 (context and reasoning) surfaces the clinical reasoning and intended care not available at each episode. The architecture is bi-directionally synchronised with existing structured databases and supports graph-native population-level analytics. Read together with Figure 2 and Table 2, the workflow proceeds from free-text clinical narratives, through NLP/LLM extraction, to graph construction and downstream research, surveillance, and planning applications.

**Figure 2:**
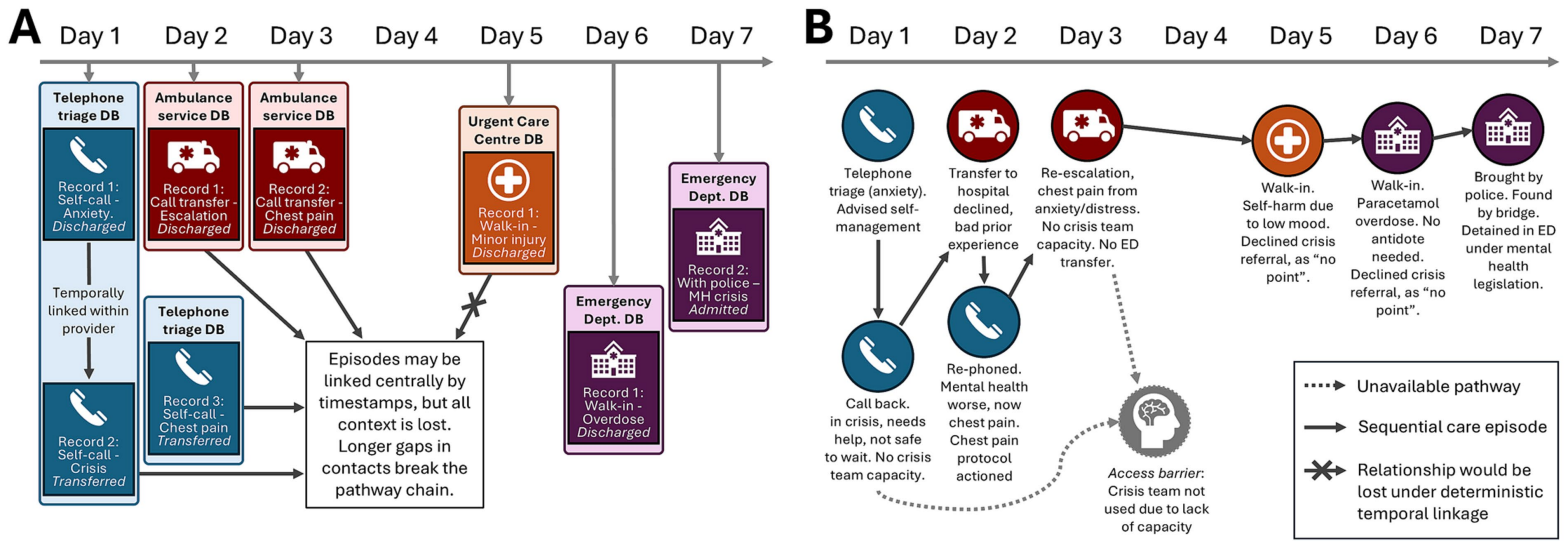
Architectural invisibility in urgent and emergency care. Panel A shows how current data architectures store the same patient’s eight contacts across four separate organisational databases as isolated, unlinked records classified by single presenting complaint codes - episodes may be linked by timestamp but all clinical context is lost. Panel B shows how a knowledge graph represents the same contacts as a connected journey, with explicit relationships between episodes and documented access barriers, making the crisis trajectory visible and queryable at population level.

We propose a layered architecture (Figure 1) in which each layer addresses a distinct data deficit and requires different technical capabilities:

Layer 1 (trajectory) links individual care contacts into connected patient journeys across services and time. Populated from existing structured, coded data - contact timestamps, patient identifiers, service codes - it is achievable as an immediate first step in systems with basic digital infrastructure. A patient experiencing mental health crisis who contacts a telephone triage service, emergency services, a walk-in facility, and an ED over 7 days (Figure 2) generates eight contacts across four services; Layer 1 represents these as a connected trajectory rather than isolated records.

Layer 2 (enriched clinical data) recovers clinical entities that structured codes fail to capture. Co-occurring psychiatric, substance use, and social dimensions are typically lost to reductive coding approaches that support only a single dominant clinical need, leaving the true complexity of presentations invisible to research and planning (10, 11). This layer extracts co-existing entities from free-text notes using established clinical NLP systems or task-specific models for well-defined entities, or large language models (LLMs) where contextual understanding is required, mapped to standardised ontologies such as SNOMED CT (18, 19).

Layer 3 (context and reasoning) captures what no coding system represents: stated reasons for presenting, barriers to care, treatment rationale, contextually inferred symptoms, and negated domains - assessments not performed, referrals not made, intended care unavailable (Table 1). This requires LLM-driven contextual inference beyond Layer 2’s entity extraction: a note recording that a clinician advised a patient to “wait until Monday” implies unavailable care without naming it; a social worker documenting that a patient “took a taxi to the ED because they have no family around” encodes social isolation without clinical terminology (20). Constrained generation approaches, where the model outputs only predefined relationship types anchored to specific text spans, make extractions auditable and limit hallucination risk, though validation against expert annotation remains essential (21, 22).

**Table 1:** Illustrative extractions from urgent and emergency care narratives across all three architectural layers.

Illustrative narrative fragment	L1: trajectory links	L2: clinical entities	L3: contextual inference (including negated domains)
“38-year-old, brought by ambulance from hostel. Lacerations to forearms, self-inflicted. Denies suicidal intent. Drinking heavily since eviction last month. Previously attended this ED 5 days ago.”	Links to ambulance conveyance record and prior ED attendance 5 days ago	Self-harm; alcohol dependence; homelessness	Symptom: suicidal intent (absent - patient denied). Presentation: presenting to ED (precipitant: eviction, inferred). Social context: escalating presentation pattern
“82-year-old, GP documented ‘no acute cause found’ for dizziness last week. Now found on floor by carer. Not eating. Recurrent falls.”	Links this ED contact to GP attendance last week in GP practice database	Frailty syndrome (beyond ICD-10 fall code); recurrent falls; nutritional neglect	Symptom: acute cause (absent - excluded by GP); functional deterioration (present - inferred from trajectory). Presentation: presenting to ED (found on floor by carer)
“Inhaler ran out, could not get GP appointment for 2 weeks. COPD flare-up. Respiratory nurse clinic cancelled last month.”	Links to cancelled respiratory clinic appointment in secondary care records	COPD exacerbation; medication supply failure	Presentation: presenting to ED/not presenting to GP (2-week wait)/not presenting to respiratory clinic (cancelled). Treatment: GP assessment (withheld - unavailable); specialist review (withheld - cancelled)
“Chest tightness since yesterday. Called 111 last night, advised to phone ambulance, but 8 h wait. On aspirin and statin since TIA last year. Pain now worsening.”	Links to 111 contact last night in 111 provider database; links to TIA admission last year in hospital records	Acute coronary risk (enriching beyond chest pain code); prior TIA; aspirin and statin (medication history)	Symptom: chest pain (present - worsening). Presentation: presenting to ED/not arriving by ambulance (8-h wait despite 111 advice). Treatment: pre-hospital assessment (withheld - ambulance delayed)

Each row shows how a single narrative fragment informs trajectory linking (L1), clinical entity extraction (L2), and contextual inference including negated domains (L3). Negated domains are clinically significant absences: assessments not performed, referrals not made, services unavailable. In deployment, each extracted relationship would carry provenance (source text span and model version) and confidence metadata; the extractions shown are illustrative examples, not outputs of a validated system.

Beyond relationship queries, knowledge graphs offer capabilities that relational architectures cannot replicate: extensibility (new data sources added without restructuring), multiple levels of representation (patient contacts linked to provider-level operational data), simultaneous analytical outputs (one graph supporting both retrospective cohort studies and real-time dashboards), and pattern discovery across connected entities (16, 17). This approach is distinct from, and complementary to, event-stream representations such as Foresight, which demonstrate that longitudinal patient event sequences provide strong predictive capability for future outcomes (23). Event streams capture what happened and when; the knowledge graph captures why, in what context, and what intended care was unavailable. The proposal also differs from existing healthcare knowledge graphs, which are typically constructed from biomedical literature, curated ontologies, or structured data, and oriented to a single condition or care setting (17, 24–26). To our knowledge, no existing framework combines cross-service trajectory linkage with a narrative-derived reasoning layer representing barriers to access and intended but unavailable care.

## Feasibility and current evidence

The feasibility of this architecture is driven by advances in transformer-based NLP. For Layer 2, the choice of extraction approach should be determined by target and documentation style; large general-purpose models are not always necessary (27). For Layer 3, however, LLMs bring a qualitatively different capability: contextual inference across the full narrative, recovering meaning that earlier approaches could not reach. LLMs have shown capability as few-shot clinical information extractors and structured clinical information extraction agents have demonstrated effectiveness in converting narrative notes into queryable data (21, 22). Extraction accuracy will vary across relationship types, with explicit barriers to access being more reliably extractable than social context requiring narrative inference (11).

Large-scale LLM-driven knowledge graph construction has been demonstrated using multicentre clinical databases, and knowledge graph-augmented retrieval has outperformed standalone language models in clinical triage (24, 28). COVID-19 knowledge graph initiatives successfully integrated heterogeneous datasets across organisational boundaries, and LLMs have been applied to extract social determinants of health from clinical narratives (25, 26, 29, 30). Foundation models trained on longitudinal clinical event sequences demonstrate capacity to predict future trajectories from sequential patterns (23).

This evidence is adjacent rather than direct. There is clear evidence that extraction of explicit clinical entities and social determinants from clinical text, knowledge graph construction from clinical databases in other settings, and trajectory prediction from coded event sequences is feasible (21, 22, 24–26, 28–30). However, we acknowledge that Layer 3 contextual inference applied to UEC documentation specifically, the three layers operating as an integrated system, or population-scale deployment remain unproven. The architecture as a whole is unvalidated, and this proposal should be read as a hypothesis whose components are individually evidenced, not as a demonstrated system. However, these converging capabilities suggest the proposed architecture is now within reach.

Crucially, perfect extraction accuracy is not a prerequisite for all use cases. Retrospective cohort analysis and systems intelligence require only data more accurate and complete than existing coded data to improve population-level inference. Proactive patient-level applications, such as real-time risk identification, require extraction credible at the individual level, demanding higher validation thresholds. This graduated requirement means the architecture can deliver value for research and population health intelligence while working towards clinical applications. Realising this potential, however, depends on governance infrastructure: systems with unified patient identifiers, cross-organisational data sharing agreements, and an institutional interest to support upstream prevention are best positioned to implement and validate the architecture first.

## Risks and limitations

Clinical narratives may encode the biases this architecture seeks to overcome. Documentation of complex patients can reflect diagnostic overshadowing, stigmatising language, and subjective behavioural judgments (31, 32). An extraction pipeline applied uncritically risks automating these artefacts into structured data. Mitigation requires extraction schemas that distinguish clinical observations from subjective interpretations, validation cohorts stratified by demographics, and involvement of patients and affected communities - not only to safeguard against bias, but to shape which questions researchers bring to the graph (33, 34).

LLMs introduce additional risks: hallucination is a documented failure mode even with constrained output approaches; outputs are sensitive to prompt formulation; and extractions may not be reproducible across model versions or updates, threatening the temporal consistency that longitudinal analysis requires (27, 35). These risks are manageable - requiring models to cite specific text spans as evidence, version-controlling prompts alongside models, and tracking model version provenance - but not eliminable. LLM extraction accuracy also varies with documentation style; the abbreviated free text of ambulance records may present challenges distinct from discharge summaries.

## From architecture to action

Three priorities determine whether this architecture delivers value or remains theoretical. The first is validation methodology specific to UEC documentation. UEC clinical notes are distinctive: abbreviated, frequently written under time pressure, spanning service-specific conventions from ambulance patient report forms to ED discharge summaries. Extraction benchmarks developed for hospital discharge letters or primary care records may not transfer directly (27). Multi-site validation studies assessing extraction accuracy across UEC documentation types, stratified by patient demographics, are needed before population-level deployment (11). Each layer implies distinct evaluation targets: for Layer 1, linkage sensitivity and specificity against gold-standard identifiers; for Layer 2, entity-level precision, recall, and F1 against expert annotation; for Layer 3, relationship-level inter-annotator agreement and the two-target validation strategy set out below. Shared annotation schemes for UEC-specific relationship types should be developed, building on existing work in social determinants extraction (29, 30). The five types used in this paper - patient_stated_reason_for_presentation, barrier_to_access, treatment_rationale, contextually_inferred_symptoms, and negated_domains (Table 1) - are our proposals for high-yield scaffold fields rather than an established schema, offered as a starting point for community refinement.

Validating Layer 3’s most distinctive output - inferred absence of intended care - poses a particular challenge: care that never happened often leaves no record against which an extraction can be checked. Two validation targets must therefore be distinguished. Fidelity to the narrative - whether the note genuinely implies the inferred absence - is testable through expert annotation with measured inter-annotator agreement, and constrained generation anchoring each inference to a quoted text span keeps every inferred absence auditable against its documentary basis. Truth about the world - whether the care was in fact unavailable - is only partially testable, but not beyond reach: some absences leave traces in other systems (cancelled clinic lists, telephone triage call logs, appointment records), permitting triangulation in a subsample and an empirical estimate of false-inference rates that bounds the error in the remainder. Pending such validation, absence inferences should carry provenance and confidence metadata, be tuned to favour precision over recall, and be restricted to population-level aggregate analysis - where bounded error rates still support valid inference about patterns of unmet need - rather than individual-level determinations.

The second validation priority is infrastructure. Cross-organisational linkage in Layer 1 depends on consistent assignment of shared patient identifiers across all services, including ambulance and walk-in settings where completeness currently falls shortest (8, 9). Data processing for Layers 2 and 3 will likely require local model deployment. Computational costs are non-trivial, reinforcing the principle that the least powerful model capable of the required accuracy should be preferred (11, 36). To give a sense of scale: a regional system serving one million people generates several hundred thousand ED attendances annually, and of the order of a million UEC contacts once ambulance, telephone triage, and out-of-hours activity are included; at roughly a thousand tokens per note, retrospective processing of a year’s narratives is of the order of a billion tokens - weeks of batch processing on a single locally hosted GPU server running a mid-sized open-weight model, rather than supercomputing scale.

Governance frameworks for LLM-derived data, including audit mechanisms detecting differential extraction accuracy across demographics, must be established before clinical deployment (33, 37). Regulatory requirements add a further gate: population-level research and planning applications sit outside medical device regulation, but the Phase 3 point-of-care uses in Table 2 would likely qualify as software as a medical device, requiring conformity assessment before deployment.

The final priority is proof-of-concept implementation targeting populations where the gap between data volume and data utility is widest and already documented (4, 10, 11). Better data architecture alone does not drive service change (38). What the knowledge graph offers is a specific remedy for a specific obstacle, making the scale and pattern of unmet need legible to decision-makers who currently lack it. The capability unlocks at each implementation phase - from trajectory linkage using existing coded data through to real-time clinical decision support - are set out in Table 2.

**Table 2:** Applications of the knowledge graph architecture, organised by cumulative implementation phase.

Implementation phase	Scenario	What the architecture enables	Current capability
L1 + existing coded data (retrospective)	Cross-system repeat attenders	Connected trajectories across organisational boundaries reveal patients cycling between services without resolution, stratified by existing diagnostic and triage codes - though code quality limits granularity	Repeat attendance counted within single provider; cross-system cycling invisible
L1 + existing coded data (retrospective)	Post-discharge re-contact sequences	Variable-length path traversal links hospital discharge to all subsequent UEC contacts across any service, revealing deterioration trajectories using existing disposition and diagnostic codes	Readmission counted as isolated event; cross-service re-contact trajectory unrecoverable
L1 + existing coded data (retrospective)	Ambulance non-conveyance outcomes	Graph traversal links each non-conveyance decision to downstream contacts across any service within 72 h, enabling outcome analysis of decisions currently made without feedback	Non-conveyance rates reported in aggregate; downstream consequences untracked
L1 + L2 + L3 enriched retrospective analysis	Primary care access failures propagating to ED	NLP-extracted triage narratives documenting failed GP access, clustered by locality and time period, linked to downstream ED trajectories - identifying upstream pressure before it reaches secondary care	ED attendance counted; upstream cause invisible
L1 + L2 + L3 enriched retrospective analysis	Systematic triage under-classification	Subgraph pattern matching across demographics identifies patient groups whose narrative acuity descriptions consistently exceed their assigned triage category, revealing classification bias invisible in coded data	Triage category recorded as fact; discrepancy between narrative and code undetectable
L1 + L2 + L3 enriched retrospective analysis	Clinician decision variation	LLM-extracted clinical reasoning enables comparison of decision logic across clinicians for matched presentations, exposing variation in reasoning rather than just variation in outcome	Outcome variation measurable; reasoning variation entirely invisible
L1 + L2 + L3 enriched retrospective analysis	Safeguarding concern accumulation	Graph algorithms detect converging low-level signals - narrative-extracted injury descriptions, attendance patterns, social context - across family members and timepoints that individually fall below reporting thresholds	Individual contacts assessed in isolation; cumulative pattern across family invisible
L1 + L2 + L3 enriched retrospective analysis	Demand surge source attribution	Graph traversal traces ED surge back through upstream trajectory nodes, with NLP-extracted patient-reported access narratives identifying specific community-level triggers	Surge measured at ED front door; upstream cause requires manual investigation
Real-time full architecture	Context-aware triage augmentation	At point of care, graph query retrieves patient’s full cross-service trajectory with extracted clinical summaries and inferred reasoning, enabling triage informed by care history no single system holds	Triage based on presenting complaint and locally available records only
Real-time full architecture	Deterioration trajectory alerting	Real-time graph pattern matching identifies patients whose current contact fits an escalating cross-service trajectory - accelerating frequency, narrowing service range, accumulating unresolved needs - triggering early intervention before crisis	Each contact assessed in isolation; escalating trajectory only recognisable retrospectively or by individual clinician recall

Phase 1 links patient trajectories across services using existing structured and coded data - already collected at population level but constrained by reductive coding and inconsistent cross-provider quality. Phase 2 adds NLP entity extraction and LLM-driven reasoning inference, enriching and correcting linked trajectories retrospectively. Phase 3 requires real-time deployment of the full architecture at point of care.

Implementation readiness varies across health system models. Internationally, integrated EHR systems consolidate data within hospital networks, but the full UEC pathway - telephone triage, ambulance, walk-in, ED, community mental health, social care - typically spans organisations with limited shared incentive or infrastructure to integrate data for upstream prevention (7, 14, 39). Even within large US health systems, Layer 1 linkage across the complete care pathway remains structurally constrained.

The architecture’s structural preconditions - unified governance, shared patient identifiers, and an institutional interest in prevention - align most fully in publicly operated health systems, though consolidated US systems such as the Veterans Health Administration share several of them. Scotland’s Unscheduled Care Datamart has linked emergency data across providers for over a decade (12) and the NHS Federated Data Platform and FHIR-based Shared Care Records are extending this integration (15, 40). Such settings offer a natural test-bed - not because the informatics problem is uniquely British, but because the governance, identifier infrastructure, and institutional motivation to act on upstream pressures are well-developed.

Where these preconditions are absent - as in much of the world’s UEC - the architecture offers less, but not nothing. Layers 2 and 3 operate on an organisation’s own clinical narratives and require no cross-organisational linkage: a single ED or ambulance service can extract clinical complexity, access barriers, and unavailable-care inferences from its own notes, gaining intelligence about the upstream pressures arriving at its doors. It is Layer 1, the connected cross-service trajectory, that fragmentation forecloses. Probabilistic linkage and regional health information exchanges offer partial substitutes, and federated approaches can preserve population-level analytics across organisational boundaries without centralised data sharing. The architecture should therefore degrade gracefully rather than failing wholesale, but its full value remains conditional on identifier and governance infrastructure that fragmented systems currently lack; evidence from integrated settings should be read with that constraint in mind.

## Conclusion

UEC generates significant volumes of data, but current architectures render most information uninterpretable for research and service planning. The three-layer knowledge graph proposed here offers a pathway from data volume to data utility: linking fragmented contacts into trajectories, recovering clinical entities from narratives, and capturing the reasoning that no structured system records. In doing so, the architecture exposes patterns of access failure and unmet need that disproportionately affect multiply disadvantaged populations - connecting informatics capability directly to health equity outcomes.

The constituent technologies now exist; the validation, governance, and infrastructure challenges are substantial but tractable. The invisibility of unmet need and unavailable care in UEC data is no longer a representational limit - it is a choice about what we build and test next.

## Data Availability

The original contributions presented in the study are included in the article/supplementary material, further inquiries can be directed to the corresponding author.
